# Cost-effectiveness of adalimumab for rheumatoid arthritis in Germany

**DOI:** 10.1007/s00393-016-0071-9

**Published:** 2016-04-14

**Authors:** C. Gissel, G. Götz, H. Repp

**Affiliations:** 1 Chair for Industrial Organization, Regulation and Antitrust, Justus Liebig University Giessen, Licher Strasse 62, 35394 Giessen, Germany; 2 Department of Internal Medicine and Rheumatology, Justus Liebig University Giessen, Giessen, Germany; 3 General Medicine, Justus Liebig University Giessen, Giessen, Germany

**Keywords:** Adalimumab, Rheumatoid arthritis, Cost effectiveness, Quality-adjusted life years, Germany, Adalimumab, Rheumatoide Arthritis, Kosten-Nutzen-Bewertung, Qualitätskorrigiertes Lebensjahr, Deutschland

## Abstract

**Background:**

In Germany, the clinical use of TNF-α inhibitors in the therapy of rheumatoid arthritis (RA) grew from 2 % of treated patients in 2000 to 20 % in 2008. In 2012, adalimumab was the bestselling drug in the statutory health insurance system with net expenditure of € 581 mio.

**Objectives:**

We aim to analyze the cost-effectiveness of adalimumab for the treatment of RA in Germany.

**Methods:**

We set up an individual patient sampling lifetime model to simulate 10,000 hypothetical patients. The patients’ functional status improves according to American College of Rheumatology response criteria. In each 6‑month cycle, treatment might be discontinued due to loss of efficacy or adverse events.

**Results:**

In the base case, patients gain 7.07 quality-adjusted life years (QALYs) with conventional synthetic therapy and 9.92 QALYs if adalimumab combination therapy is added to the treatment algorithm. The incremental cost-utility ratio (ICUR) is € 24,492 based on German list prices. After deducting mandatory rebates and taxes, the ICUR is € 17,277, comparing favorably to analyses in other countries. Adalimumab combination therapy lowers indirect costs from € 162,698 to € 134,363. The ICUR based on total costs is € 14,550 (€ 7,335 after deducting taxes and rebates). Sensitivity analysis shows that adalimumab combination therapy becomes a dominant treatment option for younger baseline populations, i. e. adalimumab is both more effective and less expensive for baseline age 30 due to savings in indirect costs.

**Conclusions:**

Our complex probabilistic model shows that estimation of cost-effectiveness for RA relies on the incorporation of indirect costs and a sufficiently long simulation horizon to capture the complete range of possible outcomes and the associated long-term benefits of biological treatment.

## Zusammenfassung

**Hintergrund:**

Der klinische Einsatz von TNF-α-Blockern in der Therapie der Rheumatoiden Arthritis (RA) ist in Deutschland von 2 % der behandelten Patienten im Jahr 2000 auf 20 % im Jahr 2008 gestiegen. 2012 lagen die Nettokosten der Gesetzlichen Krankenversicherung für Adalimumab (ADA) bei € 581 Mio.

**Ziel der Arbeit:**

Wir wollen das Kosten-Nutzen-Verhältnis von ADA in der RA-Therapie in Deutschland analysieren.

**Methoden:**

Ein Lebenszeitmodell simuliert die individuellen Krankheitsverläufe von 10.000 virtuellen Patienten mit RA anhand ihres Funktionsstatus auf Basis der Ansprechkriterien des American College of Rheumatology in sechsmonatigen Zyklen.

**Ergebnisse:**

Im Basisfall gewinnen die Patienten 7,07 qualitätskorrigierte Lebensjahre (QALYs) mit konventioneller Arzneitherapie und 9,92 QALYs mit ADA-Kombinationstherapie. Das inkrementelle Kosten-Nutzen-Verhältnis (ICUR) beträgt € 24.492 auf Basis der deutschen Listenpreise. Nach Abzug von Zwangsrabatten und Steuern liegt das ICUR bei € 17.277, was im internationalen Vergleich günstig erscheint. Durch die ADA-Kombinationstherapie sinken die indirekten Kosten von € 162.698 auf € 134.363. Das ICUR auf Basis der Gesamtkosten beträgt € 14.550 (€ 7335 nach Abzug von Zwangsrabatten und Steuern). Die Sensitivitätsanalyse zeigt, dass die ADA-Kombinationstherapie bei jungen Patienten mit einem Durchschnittsalter von 30 Jahren bei Behandlungsbeginn sowohl effektiver als auch insgesamt günstiger ist.

**Diskussion:**

Unser komplexes Wahrscheinlichkeitsmodell zeigt, dass die Berücksichtigung indirekter Kosten und ein hinreichend langer Simulationszeitraum für die Analyse des Kosten-Nutzen-Verhältnisses der RA-Arzneitherapie besonders wichtig sind, um die komplette Bandbreite möglicher Verläufe und positive Langzeiteffekte des Biologikaeinsatzes erfassen zu können.

## Introduction

Rheumatoid arthritis (RA) is the most common chronic, progressive inflammatory systemic autoimmune disease. Synovial inflammation and effusion lead to destruction of articular cartilage and joint erosion. Patients’ ability to perform daily activities can be seriously affected by joint destruction.

The overall prevalence of inflammatory arthritis is estimated at 3.4 % for the German population. The lifetime prevalence of RA is 2.5 % in Germany. RA is more common in women (3.2 %) than in men (1.9 %) [1]. RA is associated with high societal costs due to work disability. Societal cost is highest for early onset of RA in a patient‘s lifetime [2]. Each year, 17 % of RA patients undergo hospitalization [3]. RA is a painful disease with a high prevalence and a high economic burden for patients, their families and society.

The therapy of RA aims at early disease control and induction of sustained remission. Successful treatment is reflected by sustained quality of life and ability to work. Quality-adjusted life years (QALYs) are an important instrument to reflect the success of therapies in chronic diseases like RA. Further, inclusion of indirect costs, which are caused by early retirement and absence from work, is important to include in cost-effectiveness analyses.

Germany is the most important market for biological agents in the European Union. While only 2 % of RA patients were treated with TNF-α inhibitors in 2000, the popularity of TNF-α inhibitors rose to 20 % in 2008 [4]. Adalimumab (ADA) was the bestselling drug in the German statutory health insurance (SHI) system with € 581 mn net expenditure in 2012 [5]. Despite its economic relevance, cost-effectiveness of ADA treatment for RA has not been analyzed for the German SHI system.

For international comparability, we deviate from German Institute for Quality and Efficiency in Health Care’s (IQWIG) efficiency frontier method [6]. We aim to analyze the cost-effectiveness of ADA treatment for RA in terms of cost per additional QALY gained. As results of cost-utility analyses from other countries vary widely, we aim to identify the main determinants of cost-effectiveness of ADA for the German context using a modeling approach.

## Model and methods

Our cost-effectiveness analysis is based on a probabilistic lifetime model, which incorporates direct and indirect costs of RA and its treatment. We set up an individual patient sampling model to simulate 10,000 hypothetical patients in the German SHI system with a cycle length of 6 months.

Baseline patient characteristics include a mean age of 54 years (σ = 12) and an average health assessment questionnaire (HAQ) score of 1.6 (σ = 0.6). Of the total, 78 % of the hypothetical patients are female. Initial age, gender and functional status correspond to patient characteristics as enrolled in the biological arm of the German biologics register RABBIT [7].

When patients start a treatment, they can achieve one of three responses according to American College of Rheumatology (ACR) criteria or fail the therapy. Effectiveness data for each possible therapy is extracted from IQWIG‘s extensive literature review, which reflects IQWIG’s requirements for effectiveness analysis in Germany [8]. All reported trials were screened for ACR response rates. For consistency, only response rates reported after 6 months of therapy were included. All treatments and their characteristics are summarized in Table 1.

**Table 1:** Summary of treatments

	MTX	O’Dell	ADA combination therapy	Associated HAQ change (normal dist)	HAQ change STDDEV
ACR20 response (%)	31	28	58	−0.44266	0.01831
ACR50 response (%)	11	15	36	−0.66795	0.02610
ACR70 response (%)	4	7	19	−0.92257	0.03201
Duration shape parameter	0.51	0.49	0.73		
Duration scale parameter	15.73	7.31	5.96		
Direct treatment costs (Q1)	74.82 €	220.34 €	5,943.26 €		
Direct treatment costs (Qn)	77.99 €	223.51 €	5,757.71 €		

**Fig. 1:**
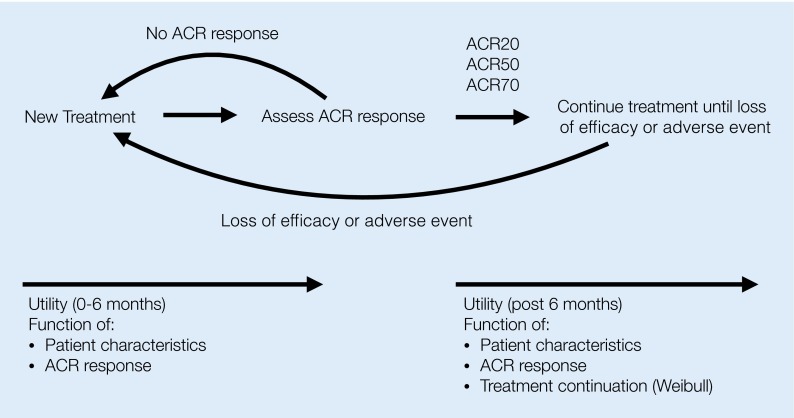
Individual patient sampling simulation approach to clinical pathways. Each patient’s response to a new treatment is measured in terms of ACR response. The patient is cycled to the next available treatment option after loss of efficacy or an adverse event

We assume each treatment in the treatment algorithm is tested for at least one period of six months, which is common in German clinical practice. If no ACR response is achieved, the patient is switched to the next treatment in the following cycle, as shown in Fig. 1. If ACR response can be achieved, we assume that each response (ACR20, ACR50 or ACR70) is associated with an initial improvement in HAQ status. We assume patients maintain their improved status throughout the course of a specific therapy.

In each cycle, treatment might be discontinued due to loss of efficacy or adverse events. We model treatment discontinuation with a Weibull distribution. Data found in the German biologicals register was not sufficient to model beyond a 6-month horizon [9]. Previously published data for Great Britain was used instead [10]. As previously suggested, functional status rebounds and patients go back to their initial functional status after failure of the therapy [11]. After failure of the last therapy in the treatment algorithm, patients are moved to a maintenance dose of MTX until the end of the overall simulation time or death.

In each cycle, quality of life is compared to hypothetical natural progression and incremental QALYs are recorded. The patient’s HAQ score is converted to quality of life using the EQ5D questionnaire. The EQ5D’s validity and reliability for RA has been described in [12]. HAQ scores are converted to EQ5D according to [13]:

$$EQ5D=0.82-0.11 \times{HAQ}-0.07\times{HAQ}^{2}$$

The *mortality risk* is calculated for each patient in each 6‑month period based on German life tables. The life tables used in our model are both age and gender specific. If the simulation results in a patient’s death during a specific modeling period, both costs and QALYs gained are logged and the next of the 10,000 patients is sampled. No influence of HAQ score is assumed on the mortality risk [14].

As suggested by German guidelines, all patients receive MTX monotherapy as first-line therapy [15]. As required for reimbursement of biological agents, patients are first switched to another conventional synthetic disease-modifying antirheumatic drug (csDMARD) therapy if MTX monotherapy fails. All patients are switched to O’Dell’s conventional synthetic triple therapy (MTX, sulfasalazine, hydroxychloroquine) after failure of first-line MTX monotherapy [16]. If triple therapy fails, patients are switched to ADA and MTX combination therapy in the biological arm or to a MTX maintenance dose in the conventional arm. No comparison to other biological agents is conducted. The model setup makes sure that all changes in effectiveness and costs can be attributed to the addition of ADA combination therapy to the treatment algorithm.

Direct cost calculations include drug costs according to the Red List 2012 and out-patient treatment costs (administration costs and screening costs before initiation of the therapy) according to German SHI out-patient payment conditions (*Einheitlicher Bewertungsmassstab, EBM*), as shown in Table 2 and 3.

**Table 2:** Direct costs (€)

	First quarter (Q1)	Following quarters (Qn)
*Methotrexate monotherapy*		
Methotrexate	24.28	27.45
Folic acid	5.98	5.98
Administration and screening	44.56	44.56
Total direct costs	74.82	77.99
*O’Dell Triple Therapy*		
Methotrexate	24.28	27.45
Folic acid	5.98	5.98
Sulfasalazine	93.67	93.67
Hydroxychloroquine	51.85	51.85
Administration and screening	44.56	44.56
Total direct costs	220.34	223.51
*Adalimumab combination therapy*		
Adalimumab	5,666.78	5,666.78
Methotrexate	24.28	27.45
Administration and screening	252.20	63.49
Total direct costs	5,943.26	5,757.71

**Table 3:** Administration and screening costs (€)

EBM code	Description	csDMARDs Q1	csDMARDs Qn	ADA Q1	ADA Qn
01321	Quarterly base rate for authorized clinics, requiring face-to-face physician-patient contact	15.77	15.77	15.77	15.77
13700	Treatment of patient with at least one additional condition: poly-oligoarthritis; seronegative ankylosing spondylitis; connective tissue disease; vasculitis; myositis			18.93	18.93
13701	Rheumatological functional diagnosis including HAQ/FFbH or DAS scores	15.95	15.95	15.95	15.95
32045	Blood sedimentation rate	0.25	0.25	0.25	0.25
32060	Blood cholesterol level			0.25	
32064	Uric acid	0.25	0.25	0.25	0.25
32065	Urea	0.25	0.25	0.25	0.25
32067	Creatinine, enzymatic	0.40	0.40	0.40	0.40
32068	Alkaline Phosphatase	0.25	0.25	0.25	0.25
32069	GOT	0.25	0.25	0.25	0.25
32070	GPT	0.25	0.25	0.25	0.25
32071	GGT	0.25	0.25	0.25	0.25
32122	Complete hemogram	1.10	1.10	1.10	1.10
32128	CRP	1.15	1.15	1.15	1.15
32823	Hepatitis B virus diagnostics			89.50	
32824	Hepatitis C virus diagnostics			89.50	
33050	Joint sonography	7.89	7.89	7.89	7.89
34220	Chest x‑ray			9.46	
40120	Mail	0.55	0.55	0.55	0.55
Sum		44.56	44.56	252.20	63.49

We extend cost computations to reflect a societal perspective. Indirect cost data according to the human capital approach is based on previously published data by the German Rheumatism Research Centre Berlin reflecting productivity losses based on its National Database of the German Collaborative Arthritis Centres, as shown in Table 4 [17, 18]. As described in detail in [17], indirect costs reflect productivity losses due to patients’ sick-leave days and permanent work disability, i. e. early retirements. Costs for sick leaves comprise the number of days of absence, which could be attributed to patients’ RA. Costs for a sick day are assumed to be equivalent to an average daily wage in Germany. Indirect costs are applied according to functional status and age, as described in [18]. All costs are discounted or inflated at an annual rate of 3 % to 2012 level, as required by IQWIG [19]. As the available published data uses the German Hannover Functional Ability Questionnaire (FFbH) rather than HAQ, the cutoff values for the functional status classes were converted to HAQ by linear transformation according to [20]:

$$HAQ=3.16-0.028\times {FFbH}$$

**Table 4:** Indirect costs according to patient age and functional status, inflated to 2012 level [17, 18]

Patient HAQ score	0.000–0.528 (in €)	0.529–1.116 (in €)	1.117–1.620 (in €)	1.621–2.096 (in €)	2.097–3.000 (in €)
Patient age < 45	766.40	4,765.59	14,775.61	21,788.45	31,119.47
Patient age 45–54	2,080.04	6,212.24	16,898.71	25,203.66	33,242.58
Patient age 55–64	5,135.49	9,944.15	18,255.42	23,314.90	28,198.30
Patient age ≥ 65	–	–	–	–	–

## Results

In the base case, patients treated with conventional synthetic therapy, on average, gain 7.07 QALYs over their lifetime. The average expected direct costs would be € 6,318, while expected total costs would be € 169,016 over a patient’s lifetime. Addition of ADA combination therapy to the treatment algorithm results in an expected lifetime gain of 9.92 QALYs. Direct costs rise to € 76,118 while overall expected costs rise to € 210,481. In the base case, the incremental cost-utility ratio (ICUR) per additional QALY gained by ADA combination therapy is € 24,492 if only direct costs are considered. The ICUR is € 14,550 if indirect costs are included, too. The base case results are summarized in Table 5. Table 6 provides a summary of the clinical pathways.

**Table 5:** Base case results

	ADA arm	csDMARD arm	Incremental
Direct costs (in €)	76,118	6,318	69,800
Indirect costs (in €)	134,363	162,698	−28,334
Mean total costs (in €)	210,481	169,016	41,465
Mean QALYs	9.92	7.07	2.85
Cost-utility (incremental cost per QALY gained)			14,550

**Table 6:** Summary of clinical pathways of 10,000 simulated patients

**Patient pathways**		**Simulation period (years)**											
*ADA sequence*		*0–0.5*	*0.5–1.0*	*1.0–1.5*	*1.5–2.0*	*2.0–2.5*	*2.5–3.0*	*3.0–3.5*	*3.5–4.0*	*4.0–4.5*	*4.5–5.0*	*5.0–5.5*	*99.5–100.0*
AdaP1	Cost	1,175.33 €	2,336.49 €	3,480.62 €	4,607.97 €	5,718.77 €	6,813.28 €	7,891.73 €	8,954.36 €	10,001.40 €	11,033.07 €	12,049.62 €	56,601.17 €
	HAQ	0.95	0.48	0.48	0.48	0.48	0.48	0.48	0.48	0.48	0.48	0.48	Dead
	QALYs	0.06	0.14	0.22	0.33	0.45	0.58	0.73	0.90	1.09	1.29	1.52	35.27
AdaP2	Cost	8,475.97 €	16,830.66 €	25,062.79 €	33,174.15 €	41,166.50 €	49,041.60 €	56,801.17 €	64,446.90 €	71,980.46 €	79,403.50 €	86,717.64 €	259,074.20 €
	HAQ	1.76	1.33	1.33	1.33	1.33	1.33	1.33	1.33	1.33	1.33	1.33	Dead
	QALYs	0.09	0.20	0.33	0.47	0.64	0.83	1.05	1.28	1.54	1.83	2.14	19.74
AdaP3	Cost	5,049.70 €	5,201.14 €	5,350.35 €	5,497.38 €	5,642.25 €	5,784.99 €	5,925.64 €	6,064.23 €	6,200.78 €	6,335.33 €	6,467.90 €	22,217.08 €
	HAQ	1.51	1.09	1.09	1.09	1.09	1.09	1.09	1.09	1.09	1.09	1.09	Dead
	QALYs	0.08	0.17	0.28	0.41	0.56	0.73	0.92	1.13	1.36	1.62	1.90	13.40
AdaP4	Cost	12,567.52 €	16,014.09 €	19,413.13 €	22,762.30 €	26,062.34 €	29,313.96 €	32,517.88 €	35,674.80 €	38,785.40 €	49,655.85 €	62,241.19 €	283,043.36 €
	HAQ	1.83	1.69	1.04	1.04	1.04	1.04	1.04	1.04	1.04	1.04	1.69	Dead
	QALYs	0.04	0.20	0.38	0.58	0.81	1.05	1.32	1.61	1.93	2.17	2.54	7.33
AdaP10000	Cost	5,049.70 €	10,028.39 €	14,934.04 €	19,767.72 €	24,530.49 €	29,223.39 €	33,847.43 €	41,957.29 €	44,593.79 €	47,194.35 €	49,756.75 €	99,881.03 €
	HAQ	1.29	0.66	0.66	0.66	0.66	0.66	0.66	0.66	1.29	0.35	0.35	Dead
	QALYs	0.09	0.20	0.32	0.46	0.62	0.80	1.00	1.13	1.40	1.68	1.99	22.02
*Mean cost (10,000 patients) = 210,481 €*													
*Mean QALYs (10,000 patients) = 9.92*													
**Patient pathways**		**Simulation period (years)**											
*csDMARD sequence*		*0–0.5*	*0.5–1.0*	*1.0–1.5*	*1.5–2.0*	*2.0–2.5*	*2.5–3.0*	*3.0–3.5*	*3.5–4.0*	*4.0–4.5*	*4.5–5.0*	*5.0–5.5*	*99.5–100.0*
ConvP1	Cost	3,211.12 €	4,651.77 €	7,623.18 €	10,698.02 €	13,727.75 €	16,713.04 €	19,654.52 €	27,300.25 €	34,833.82 €	42,256.85 €	49,570.99 €	256,125.65 €
	HAQ	0.95	0.81	0.36	0.81	0.88	0.94	1.01	1.07	1.14	1.20	1.27	Dead
	QALYs	0.03	0.11	0.17	0.23	0.30	0.38	0.46	0.56	0.66	0.78	0.90	4.57
ConvP2	Cost	8,475.97 €	11,922.54 €	15,321.58 €	18,670.75 €	21,970.79 €	25,222.41 €	28,426.33 €	31,583.24 €	34,693.85 €	37,758.81 €	40,778.81 €	222,335.27 €
	HAQ	1.76	1.62	1.00	1.00	1.00	1.00	1.00	1.00	1.00	1.00	1.00	Dead
	QALYs	0.04	0.20	0.37	0.56	0.78	1.01	1.27	1.55	1.86	2.19	2.54	9.61
ConvP3	Cost	5,049.70 €	5,201.14 €	5,350.35 €	5,497.38 €	5,642.25 €	5,642.25 €	6,042.47 €	6,181.06 €	6,317.61 €	6,452.16 €	6,584.74 €	7,459.88 €
	HAQ	1.51	1.02	1.02	1.02	1.02	1.02	1.51	1.36	1.43	1.49	1.56	Dead
	QALYs	0.09	0.19	0.31	0.45	0.61	0.72	0.87	1.03	1.21	1.39	1.59	3.21
ConvP4	Cost	12,567.52 €	21,201.70 €	33,256.99 €	45,282.45 €	57,131.49 €	68,806.69 €	80,310.61 €	91,645.76 €	102,814.61 €	117,286.83 €	131,546.73 €	364,983.07 €
	HAQ	1.83	1.68	1.24	1.68	1.75	1.81	1.88	1.94	2.01	2.07	2.14	Dead
	QALYs	0.04	0.18	0.26	0.35	0.46	0.57	0.69	0.83	0.97	1.13	1.30	2.40
ConvP10000	Cost	9,144.37 €	14,402.55 €	19,308.20 €	28,058.97 €	36,681.36 €	45,177.25 €	53,548.50 €	61,796.94 €	69,924.37 €	77,932.57 €	85,823.28 €	118,358.40 €
	HAQ	1.29	1.17	1.03	1.10	1.16	1.23	1.29	1.36	1.42	1.49	1.55	Dead
	QALYs	0.03	0.10	0.17	0.25	0.34	0.44	0.55	0.67	0.80	0.94	1.09	3.96
*Mean cost (10,000 patients) = 169,016 €*													
*Mean QALYs (10,000 patients) = 7.07*													

While no ICUR threshold has been defined for Germany, € 60,000 per QALY gained is a value that is known to be accepted for treatments by the SHI funds in Germany. This value has been suggested for cost-utility analysis of biological agents in Germany [21]. The ICUR for the base-case is well below this threshold for both direct and total costs per QALY gained.

The results reported for the base case potentially overestimate German ICURs for international comparison. In contrast to other countries like Sweden or the United Kingdom, German pharmaceutical list prices are distorted by inclusion of the full value-added tax (VAT) of 19 % and a mandatory rebate, which is reimbursed by the manufacturer to the SHI funds. Taxes are used to subsidize the German SHI funds on a regular basis. The mandatory rebate is subject to the political decision making process. It frequently changes with new government coalitions. For the purpose of our analysis, we assume a 16 % mandatory rebate, which has been applied from August 2010 to December 2013.

For the base case, direct costs are only € 54,507 for ADA combination therapy and € 5,269 for conventional monotherapy if VAT and mandatory rebates are excluded from cost calculations. Adjusted ICURs are € 17,277 for direct costs and € 7,335 for total costs.

## Sensitivity analysis

Results of cost-effectiveness analyses for biological agents vary greatly, to some extent due to different assumptions in the underlying models. We analyze the impact of various changes in our model parameters. For international comparison, all results of the sensitivity analysis are reported with VAT/rebate-adjusted results in brackets.

We test the impact of changes in patient characteristics. We alter baseline age to 30 years, 40 years and 60 years. We alter initial HAQ score to 1.0, 2.0 and 2.5. We further change the discount rate of all costs to 0 % and to 6 %. Additionally, we introduce a 3 % discount rate for QALYs gained as suggested in [22]. We limit the modeling period to 5 years and to 10 years instead of the base case’s lifetime perspective. All results are summarized in Table 7.

**Table 7:** Deterministic sensitivity analysis

			ADA	ADA	csDMARDs	csDMARDs	ICUR ADA	ICUR ADA	ICUR change compared to base case		% Change compared to base case	
			Retail price	Undistorted price	Retail price	Undistorted price	Retail price	Undistorted price	Retail price	Undistorted price	Retail price	Undistorted price
BASE CASE	Costs	Direct	*76,118 €*	*54,507 €*	*6,318 €*	*5,269 €*	*24,492 €*	*17,277 €*				
		Total	*210,481 €*	*188,870 €*	*169,016 €*	*167,967 €*	*14,550 €*	*7,335 €*				
	QALYs		*9.92*	*9.92*	*7.07*	*7.07*						
Baseline age 30	Costs	Direct	95,238 €	68,368 €	9,046 €	7,590 €	19,804 €	13,965 €	−4,688 €	−3,312 €	−19 %	−19 %
		Total	444,974 €	418,105 €	429,773 €	428,317 €	3,493 €	−2,346 €	−11,057 €	−9,681 €	−76 %	−132 %
	QALYs		14.90	14.90	10.55	10.55						
Baseline age 40	Costs	Direct	92,343 €	66,182 €	8,092 €	6,775 €	22,778 €	16,061 €	−1,713 €	−1,215 €	−7 %	−7 %
		Total	356,685 €	330,524 €	329,333 €	328,016 €	7,395 €	678 €	−7,155 €	−6,657 €	−49 %	−91 %
	QALYs		13.06	13.06	9.36	9.36						
Baseline age 60	Costs	Direct	67,358 €	48,211 €	5,468 €	4,550 €	26,137 €	18,439 €	1,645 €	1,162 €	7 %	7 %
		Total	151,573 €	132,427 €	108,217 €	107,300 €	18,310 €	10,612 €	3,760 €	3,277 €	26 %	45 %
	QALYs		8.36	8.36	5.99	5.99						
Baseline HAQ 1.0	Costs	Direct	75,076 €	53,771 €	6,269 €	5,229 €	26,010 €	18,350 €	1,518 €	1,073 €	6 %	6 %
		Total	165,843 €	144,538 €	123,945 €	122,905 €	15,838 €	8,178 €	1,288 €	843 €	9 %	11 %
	QALYs		10.72	10.72	8.07	8.07						
Baseline HAQ 2.0	Costs	Direct	76,607 €	54,853 €	6,285 €	5,245 €	26,281 €	18,540 €	1,789 €	1,263 €	7 %	7 %
		Total	245,383 €	223,629 €	198,697 €	197,656 €	17,448 €	9,707 €	2,898 €	2,372 €	20 %	32 %
	QALYs		8.67	8.67	5.99	5.99						
Baseline HAQ 2.5	Costs	Direct	76,869 €	55,033 €	6,278 €	5,237 €	31,950 €	22,538 €	7,459 €	5,261 €	30 %	30 %
		Total	282,295 €	260,458 €	226,992 €	225,951 €	25,031 €	15,618 €	10,481 €	8,284 €	72 %	113 %
	QALYs		6.78	6.78	4.57	4.57						
5-year results	Costs	Direct	27,429 €	19,628 €	1,934 €	1,582 €	86,438 €	61,185 €	61,947 €	43,908 €	253 %	254 %
		Total	71,005 €	63,205 €	53,749 €	53,397 €	58,503 €	33,250 €	43,954 €	25,915 €	302 %	353 %
	QALYs		1.65	1.65	1.35	1.35						
10-year results	Costs	Direct	50,187 €	35,836 €	3,377 €	2,782 €	52,128 €	36,810 €	27,636 €	19,534 €	113 %	113 %
		Total	130,019 €	115,669 €	101,776 €	101,180 €	31,452 €	16,135 €	16,902 €	8,800 €	116 %	120 %
	QALYs		4.19	4.19	3.29	3.29						
0 % cost discounting	Costs	Direct	107,825 €	77,278 €	9,385 €	7,867 €	33,740 €	23,790 €	9,248 €	6,513 €	38 %	38 %
		Total	290,850 €	260,302 €	231,072 €	229,554 €	20,488 €	10,539 €	5,939 €	3,204 €	41 %	44 %
	QALYs		9.98	9.98	7.06	7.06						
6 % cost discounting	Costs	Direct	59,773 €	42,763 €	4,683 €	3,888 €	20,844 €	14,709 €	−3,648 €	−2,568 €	−15 %	−15 %
		Total	163,092 €	146,082 €	130,289 €	129,494 €	12,411 €	6,276 €	−2,138 €	−1,058 €	−15 %	−14 %
	QALYs		9.89	9.89	7.24	7.24						
3 % QALY discounting	Costs	Direct	76,659 €	54,887 €	6,287 €	5,247 €	37,726 €	26,612 €	13,235 €	9,335 €	54 %	54 %
		Total	208,460 €	186,688 €	166,432 €	165,392 €	22,531 €	11,417 €	7,982 €	4,082 €	55 %	56 %
	QALYs		6.84	6.84	4.98	4.98						
HUI-3 for quality of life	Costs	Direct	76,228 €	54,583 €	6,319 €	5,271 €	29,678 €	20,934 €	5,186 €	3,657 €	21 %	21 %
		Total	209,029 €	187,384 €	168,860 €	167,812 €	17,053 €	8,309 €	2,503 €	974 €	17 %	13 %
	QALYs		7.41	7.41	5.05	5.05						
Direct costs +10 %	Costs	Direct	83,730 €	59,957 €	6,950 €	5,796 €	26,941 €	19,004 €	2,449 €	1,728 €	10 %	10 %
		Total	218,093 €	194,321 €	169,647 €	168,494 €	16,999 €	9,062 €	2,449 €	1,728 €	17 %	24 %
	QALYs		9.92	9.92	7.07	7.07						
Direct costs −10 %	Costs	Direct	68,506 €	49,056 €	5,686 €	4,742 €	22,043 €	15,549 €	−2,449 €	−1,728 €	−10 %	−10 %
		Total	202,869 €	183,419 €	168,384 €	167,440 €	12,100 €	5,607 €	−2,449 €	−1,728 €	−17 %	−24 %
	QALYs		9.92	9.92	7.07	7.07						
Indirect costs +10 %	Costs	Direct	76,118 €	54,507 €	6,318 €	5,269 €	24,492 €	17,277 €	0 €	0 €	0 %	0 %
		Total	223,917 €	202,306 €	185,285 €	184,236 €	13,555 €	6,340 €	−994 €	−994 €	−7 %	−14 %
	QALYs		9.92	9.92	7.07	7.07						
Indirect costs −10 %	Costs	Direct	76,118 €	54,507 €	6,318 €	5,269 €	24,492 €	17,277 €	0 €	0 €	0 %	0 %
		Total	197,045 €	175,434 €	152,746 €	151,697 €	15,544 €	8,329 €	994 €	994 €	7 %	14 %
	QALYs		9.92	9.92	7.07	7.07						

The biggest increase in ICURs can be seen by limiting the simulation period. If the maximum simulation period is limited to 10 years, ICURs double. The effect is even bigger for a limit of 5 years. ICURs for direct costs only rise to € 86,438 (€ 61,185). ICURs for total costs rise to € 58,503 (€ 33,250). This finding emphasizes that a longer time horizon is necessary to capture the long-term benefits of biological therapy as a treatment option after the failure of csDMARDs.

The magnitude of ICUR changes caused by discounting changes implies that careful attention needs to be paid to discounting assumptions, when comparing results among different models.

Different scenarios for the average age of the hypothetical population have a small effect on ICURs if only direct costs are considered. Baseline age effect increases if indirect costs are included in the cost-utility analysis. For baseline age 30, ICURs based on gross costs decrease by 76 % to € 3,493. If rebates and VAT are deducted, ADA is a dominant treatment, i. e. patients gain more QALYs (14.90 vs. 10.55) at lower total costs (€ 418,105 vs. € 428,317).

Total costs rise with baseline HAQ. Fewer QALYs are gained and patients remain in a bad functional state, which prevents them from working. ICURs were slightly worse for a baseline HAQ of 1.0 instead of the base case’s 1.6, perhaps indicating that patients with a HAQ score of 1.0 cannot benefit as much from biological therapy as patients with a HAQ score between 1.0 and 2.0.

If quality of life is not calculated by conversion of HAQ scores to EQ5D but by a different questionnaire, the HUI-3 as proposed in [23], ICURs rise.

ICURs rise and fall with direct costs and even more so if ICURs are based on total costs. If indirect costs rise, the ICUR for ADA combination therapy falls and vice versa. This explains why ICURs in Germany are fairly low despite high list prices for ADA. In lower-income countries like Colombia, the ICUR for biological therapy has been reported to be € 137,723 due to much lower indirect costs [24].

**Fig. 2:**
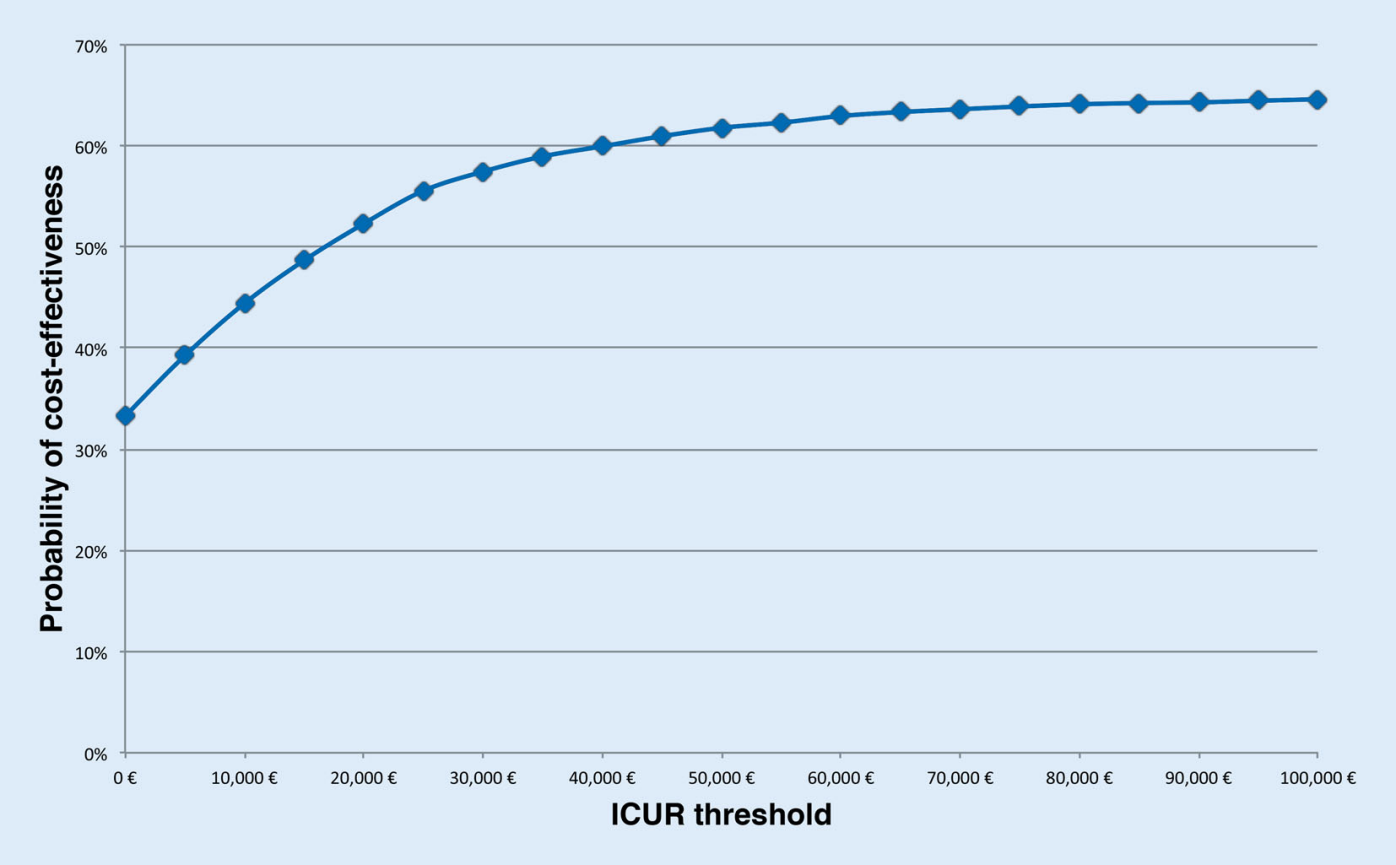
Cost-effectiveness acceptability curve for the base case scenario. The curve shows the percentage of simulated patients, whose treatment with adalimumab combination therapy would fulfill a specific incremental cost-utility ratio (ICUR), which the regulator can define

Fig. 2 shows the cost-effectiveness acceptability curve (CEAC) for the base case. The CEAC is an important decision tool for the regulator to measure the uncertainty associated with accepting ADA therapy at a specific ICUR threshold [25]. Even for the most restrictive threshold, i. e. € 0.00 per additional QALY gained, the treatment of more than 30 % of the simulated population would be cost-effective.

Our sensitivity analysis helps to identify patient subgroups that belong to the 30 % of cost-effectively treated patients. The sensitivity analysis suggests young patients can be treated most cost-effectively, because their direct cost increases are overcompensated by indirect cost savings.

The individual sampling approach shows that ADA therapy will not meet the threshold for 35 % of patients even if the threshold is set is as high as € 100,000, i. e. some patients might incur high costs under ADA therapy without benefiting from the therapy in a way that would be considered cost-effective.

## Discussion and conclusions

Despite ADA’s clinical and economic relevance over the last years, our study is the first one to assess its cost-effectiveness for the German SHI system. We could only identify one previously published cost-effectiveness analysis for a TNF-α inhibitor for RA in Germany [18]. Cost per QALY gained is estimated at € 38,700 Euro for etanercept combination therapy. The study was conducted on a 10-year time horizon, including indirect costs. Other studies analyzed second-line biologic agents after the failure of a TNF-α inhibitor [26, 27]. Only one reported incremental cost-per-QALY ratios. Adding rituximab to the treatment algorithm after failure of etanercept resulted in an ICUR of € 24,517 for direct costs only. The ICUR was only € 15,565 if indirect costs were included [27].

The results of our analysis suggest that ADA is a cost-effective biological agent, which is beneficial to the patient and society as a whole, when used after the failure of conventional therapy. Multiple factors contribute to ADA’s cost-effectiveness in Germany. Clinical evidence shows ADA’s superior effectiveness after failure of MTX when used as a combination therapy [28]. Our model reflects this finding with higher QALY gains in the biological arm. ADA’s effectiveness often prevents long-term loss of work capacity, when the patient is at high risk after the failure of csDMARDs.

This is further emphasized by the finding that ADA becomes more cost-effective for younger populations, i. e. populations who have more time left until retirement. In addition to population age, derivation of quality of life from functional status and discounting of future QALYs gained are decisive factors for the cost-effectiveness of ADA combination therapy. This should be kept in mind when designing cost-effectiveness models for biological treatments for RA.

Even if only direct costs are considered, the ICUR found in our analysis for ADA combination therapy (€ 24,492) compares favorably to results found for Sweden (€ 34,922), Great Britain (£ 34,300) and China ($ 57,926) [23, 29, 30]. However, international comparison of results remains difficult due to differences in methodology, even though the same measure of cost-effectiveness is used, i. e. cost per additional QALY gained.

Our analysis has shown that ADA combination therapy is cost-effective by all known standards for the German SHI system. Cost-effectiveness is heavily influenced by indirect costs because of RA’s influence on the patients’ ability to work. For a very young population (baseline age 30), direct costs incurred by biological treatment are overcompensated by indirect cost savings at a higher quality of life for the patient.

Due to the lack of head-to-head comparisons of biological agents, further modeling approaches are needed to compare the cost-effectiveness of different biological agents for the German market. Further opportunities might arise by earlier use of biological agents before the failure of multiple conventional therapies. If a window of opportunity exists in early RA, use of biological agents as first-line therapy could be cost-effective in the long-term, especially for a young population.
